# Regulation of aging by balancing mitochondrial function and antioxidant levels

**DOI:** 10.1186/s12576-022-00853-1

**Published:** 2022-11-15

**Authors:** Sawako Yoshina, Luna Izuhara, Naoyuki Kamatani, Shohei Mitani

**Affiliations:** 1 Department of Physiology, Tokyo Women’s Medical University School of Medicine, 8-1, Kawada-Cho, Shinjuku-Ku, Tokyo, 162-8666 Japan; 2 StaGen Co., Ltd, 4-11-6, Kuramae, Taito-Ku, Tokyo, 111-0051 Japan; 3 Institute for Comprehensive Medical Sciences, Tokyo Women’s Medical University, 8-1, Kawada-Cho, Shinjuku-Ku, Tokyo, 162-8666 Japan

**Keywords:** Febuxostat, Aging, Xanthine oxidase (XO), Xanthine dehydrogenase (XDH), ATP, *C. elegans*

## Abstract

Aging is the deterioration of physiological mechanisms that is associated with getting old. There is a link between aging and mitochondrial function. However, there is an unresolved relationship between ATP levels and aging. To address this issue, we administered febuxostat (FBX), an inhibitor of human xanthine oxidase (XO)/xanthine dehydrogenase (XDH), to *C. elegans*. We used *C. elegans* as a model to evaluate the effects of FBX and to challenge the enigma of the relationship between ATP and lifespan. In this study, we showed that FBX protects mitochondria and prevents age-related muscle deterioration in *C. elegans.* In addition, we showed that FBX administration could increase ATP levels without overloading the mitochondria while extending the lifespan. We also showed that the combination of FBX and an antioxidant as a protection against ROS prolongs lifespan more. We have shown that the antioxidant effects and increased ATP levels may lead to antiaging effects.

**Supplementary Information:**

The online version contains supplementary material available at 10.1186/s12576-022-00853-1.

## Background

Aging is the deterioration of physiological mechanisms that is associated with getting old. Aging is closely related to age-related diseases and human beings’ death. In humans, aging is accompanied by hearing impairment, vision deterioration, muscle mass reduction, respiratory capacity reduction, and organ weight decrease. The accumulation of DNA damage, mitochondrial damage, and damaged proteins are believed to cause aging [1–3]. Radiation, ultraviolet rays, and reactive oxygen species are known to cause the accumulation of DNA damage [4]. The mitochondrial electron transfer system produces ROS. The accumulation of mitochondrial damage is also believed to involve ROS. The antioxidant defense system normally works to protect target molecules from ROS in the mitochondria of normally functioning young cells. However, the activity and expression of antioxidant enzymes are decreased in the mitochondria of aged cells, and these decreases are believed to lead to the accumulation of oxidative damage [5, 6]. It has been suggested that reducing ROS can improve aging and age-related diseases in model organisms [7–9]. In humans, uric acid, vitamin C, and vitamin E exert antioxidant effects on ROS. Uric acid is the end product of purine metabolism in humans and is one of the most abundant antioxidant molecules [10]. Uric acid is known to extend lifespan by activating DAF-16/FOXO and SKN-1/NRF2 in *C. elegans* [11].

In *C. elegans*, it was reported that inhibiting the electron transfer system decreases ATP production and extends lifespan and that suppressing oxygen consumption extends lifespan [12–16]. However, it has also been suggested that energy loss due to mitochondrial dysfunction or impairment is a mechanism of diseases and aging [17–19]. In *C. elegans*, it was reported that mutants of *clk-1*, *3*, *5*, *6*, and *10* demonstrated increased ATP levels and longer lifespans [7]. Thus, there are apparent contradictory hypotheses regarding mitochondrial function, ATP levels, and lifespan.

Additionally, in mice, *C. elegans*, and flies, calorie restriction suppresses insulin signaling, activates FOXO, and extends lifespan [20, 21]. The inhibition of insulin receptor/daf-2 expression in *C. elegans* has been reported to increase ATP levels and prolong lifespan [14]. Although there are reports on genes involved in lifespan as described above, no treatment has been found to reliably suppress aging. Furthermore, the relationship between ATP levels and lifespan is still elusive.

In this study, we administered febuxostat (FBX), an inhibitor of human xanthine oxidase (XO)/xanthine dehydrogenase (XDH), to *C. elegans*. XO/XDH is an enzyme that participates in the in vivo synthesis of uric acid. When FBX is added to cells, the salvage pathway of purine metabolism becomes dominant, and the ATP concentration increases [17, 22]. It is also known that inhibitors of XO/XDH prevent the onset of heart disorders and dementia, similar to aging [23, 24]. Additionally, inhibitors of XO/XDH were reported to partially prevent disuse muscle atrophy in mice and humans [25]. Most mammals, including mice, have uricase, which breaks down uric acid. However, humans cannot breakdown uric acid because of inactive urate oxidase gene. Since *C. elegans* also lacks uricase [26, 27], we used *C. elegans* as a model to evaluate the inhibitory effect of XO/XDH and challenged the enigma of ATP–lifespan relationships.

## Materials and methods

### Nematode strains

*Caenorhabditis elegans* strain N2 worms were used as wild-type animals. Worms were grown at 20 °C under well-fed conditions using standard methods [28]. The strain carrying *hprt-1*(*tm6318)* was obtained from a UV/TMP-mutagenized library as described previously [29]. These were identified via PCR amplification with primers spanning the deletion region of *tm6318*, as described previously [29, 30]. The primers used for PCR genotyping were as follows: 5′-CAATCGCGCTGCTCTGCGTA-3′ and 5′-CTATACTGGCAAAACGCGGT-3′ (*tm6318* 1st round); 5′-GCGTACTCAAAGGATCCTAT-3′ and 5′-GACGGTCATAATACACCGAA-3′ (*tm6318* 2nd round). The mutant was backcrossed twice with N2 before use. *tm6318* is a mutant that partially lacks the phosphoribosyl transferase domain.

Strains carrying the *xdh-1* mutation (*tm9909* and *tm9911*) were obtained from a CRISPR–cas9 system as described previously [31] and identified via PCR amplification with primers spanning the deletion region of *tm9909* and *tm9911.* The primers used for PCR genotyping were 5′-GAGTGCAAGACTAATAGGGAG-3′ and 5′-GTGTTTCACCCCTTCTCTAG-3′. The mutants were backcrossed twice with N2 before use. *tm9909* and *tm9911* are mutants lacking almost all coding sequences.

The Caenorhabditis Genetics Center provided the *xdh-1(ok3234)* mutant animals. All assays were performed on kanamycin (Km)-supplemented NGM plates with UV-irradiated OP50 as the food, unless otherwise indicated. UV treatment of bacteria was prepared as described previously [32].

### Plasmid construction

We used site-directed mutagenesis to insert the guide sequences into a Cas9-sgRNA (single guide RNA) expression vector (pDD162) containing both sgRNA and Cas9 protein expression units, which were obtained through Addgene [33]. Then, *xdh-1* was targeted for Cas9 cleavage using the guide sequences (GAATACGTTCAGGAGTTGC and GATGCAATGAGGGAGGATG) that were inserted into pDD162 individually.

### Movement analyses

Eggs were collected by bleaching transgenic (Tg) animals (*tmIs388* or *tmIs390*) reared at 20 °C on OP50 normally seeded NGM plates (Day 0). After 36 h, bleached Tg animals were transferred to NGM plates with FBX. OP50 was irradiated with UV and treated with Km. When the Tg animals reached the young adult age, FUdR (15 μM) was added to the NGM plate. Eleven days after bleaching, a Tg animal expressing Tau was placed on a new NGM plate with one animal each. After 30 min, we photographed the traces of worm movement using a stereomicroscope (Olympus). The areas with worm movement were quantified using ImageJ (NIH, Bethesda, MD). At least 30 animals were observed per condition at a time. The experiments were repeated four times.

### Growth analysis of S129A-α-synuclein Tg worms

We used *C. elegans* overexpressing Ser-129 mutant α-synuclein and EGFP (α-Syn [S129A], *tmIs913*) or only EGFP (*tmIs907*) as a control. α-Syn (S129A) and EGFP were expressed under the *unc-51* promoter, which drives the pan-neuronal expression. α-Syn (S129A) Tg worms exhibit growth retardation [34].

Eggs were collected by bleaching Tg animals (*tmIs913* or *tmIs907*) reared at 20 °C on OP50 normally seeded NGM plates. Then, 36 h after bleaching, Tg animals were transferred to NGM plates with FBX. OP50 was irradiated with UV and treated with Km. Then, 96 h after bleaching, we individually photographed the fluorescence of the worms using a fluorescence stereomicroscope (Olympus). The glowing area was measured using ImageJ (NIH, Bethesda, MD). At least 30 animals were observed per condition at a time. The experiments were repeated four times.

### Transmission electron microscopy

TEM analyses of *C. elegans* were performed as previously described [35]. For synchronized worms, eggs were obtained by bleaching gravid N2 adult hermaphrodites (Day 0). These eggs were reared at 20 °C on OP50 normally seeded NGM plates. Then, 36 h after bleaching, worms were transferred to NGM medium with FBX (0, 5, 10, or 20 μg/ml). OP50 was irradiated with UV and treated with Km. After Day 4, we replanted every day until the worm stopped laying eggs. On Day 18, we fixed the worm. In brief, the worms were anesthetized in M9 buffer with 8% ethanol for 5 min and then cut into 2–3 pieces in the primary fixative solution (2% glutaraldehyde, 2% paraformaldehyde in 100 mM phosphate buffer, pH 7.4). Then, they were postfixed in osmium tetroxide at 4 °C, dehydrated, and embedded in EPON812. The posterior side of the vulva was used as a sample. Ultrathin sections were analyzed using a transmission electron microscope (HITACHI H-7600) at 100 keV. TEM analyses were performed at the Hanaichi Ultrastructure Research Institute Co. (Okazaki, Japan).

### Lifespan analysis

For synchronized worms, eggs were obtained by bleaching gravid adult hermaphrodites (Day 0). These eggs were reared at 20 °C on OP50 normally seeded NGM plates. Then, 36 h after bleaching, the Tg animals were transferred to NGM plates with FBX (0, 5, 10, or 20 μg/ml) and vitamin C (0 or 4 mM). OP50 was irradiated with UV and treated with Km (20 µg/ml). The UV treatment of bacteria was prepared as described previously [32].

Upon reaching young adult age, worms were supplied with FUdR (15 μM). Lifespan measurements were initiated by the transfer of adult-stage worms (Day 5) to new NGM plates containing 5-fluoro-2′-deoxyuridine (FUdR, 15 μM), FBX (0, 5, 10, or 20 μg/ml) and vitamin C (0 or 4 mM). OP50 was irradiated with UV and treated with Km (20 µg/ml). Worms were transferred to fresh plates every 7 days. To prevent progeny development, plates were supplemented with FUdR (15 μM) on the day before use. Survival was monitored every other day; worms were considered dead if they showed no movement when prodded with a platinum wire. Lifespan analysis via log-rank tests was performed using GraphPad Prism 6.

### ATP detection

The assessment of the ATP levels of *C. elegans* was performed as previously described [36]. In brief, N2 worms (approximately 50 animals each) in the L4 stage were placed on NGM plates supplemented with 500 µM NaN_3_, FBX (0 or 20 µg/ml), and sodium ascorbate (Vit. C, 0 or 4 mM). After 14 h, only adult nematodes were collected and washed 5 times with M9 buffer. The samples were frozen using liquid nitrogen, and then the frozen worm was boiled for 15 min. The samples were centrifuged at 14,800 *g* for 10 min at 4 °C, and then the supernatants were transferred into new 1.5-ml tubes. ATP was determined using the ATP Determination Kit (A22066, Molecular Probes) according to the manufacturer’s protocol.

### mtDNA content quantification

The mitochondrial DNA (mtDNA) copy number was quantified using real-time quantitative PCR (qPCR). N2 worms (approximately 50 animals each) in the L4 stage were placed on NGM plates supplemented with 500 µM NaN_3_, FBX (0 or 20 μg/ml), and sodium ascorbate (0 or 4 mM). After 14 h, only adult nematodes were collected and lysed using proteinase K. Then, qPCR was performed using SYBR GREEN PCR Master Mix (Applied Biosystems) with the *nd-1* forward (5-AGCGTCATTTATTGGGAAGAAGAC-3) and reverse (5-AAGCTTGTGCTAATCCCATAAATGT-3) primers, as well as the *ama-1* forward (5-AGATGGACCTCACCGACAAC-3) and reverse (5-CTGCAGATTACACGGAAGCA-3) primers. The mtDNA copy number was calculated as the amplification of the mitochondrial gene (*nd-1*) relative to the amplification of the nuclear gene (*ama-1*). The experiments were repeated nine times.

### Assay for NaN_3_ resistance

*C. elegans* of the L4 stage were placed on NGM plates, which were supplemented with NaN_3_ (400 or 500 μM), FBX (0, 5, 10, or 20 μg/ml), sodium ascorbate (0 or 4 mM), uric acid (0 or 2 mM), and Km. UV-irradiated OP50 was fed to the animals. After 8, 14, 20, and 32 h, we observed the survival of *C. elegans*.

### Uric acid quantity measurement

For synchronized worms, eggs were obtained by bleaching gravid N2 or *tm9911* worms (Day 0). After 36 h, the worms were transferred to NGM plates with FBX (0, 5, 10, or 20 μg/ml). UV-irradiated OP50 was used as food. Worms were supplied with FUdR (15 μM) on Day 4. On Day 6, adult worms were collected for the preparation of urate samples as described previously [26]. In brief, ~ 500 worms in the experimental or control groups were collected, washed with M9 buffer three times, and ultrasonicated. Protein was precipitated by centrifugation (4000 rpm for 20 min), and then the supernatant was analyzed using the Uric Acid Assay Kit (CBL, STA-375) according to the manufacturer’s protocol.

### Mitochondrial imaging and nuclear imaging

*ccIs4251 (Pmyo-3::Ngfp-lacZ; Pmyo-3::Mtgfp)*, which has GFP fusion proteins localized to the body wall muscle mitochondria and nuclei, was used for this study. For synchronized worms, eggs were collected by bleaching Tg animals (*ccIs4251*) and reared at 20 °C on OP50 normally seeded NGM plates (Day 0). After 36 h, the worms were transferred to NGM plates with FBX (0, 5, 10, or 20 µg/ml) and Km. UV-irradiated OP50 was used as food. After Day 4, we replanted every day until the worm stopped laying eggs. On Days 14, 16, and 18 after bleaching, worms were anesthetized by placing M9 buffer with a drop of 50 mM sodium azide on the solidified pads of 5% agarose laid on the slides. After adding a coverslip, worms were observed using a BX-51 microscope (Olympus). Mitochondrial morphology was classified in accordance with previous studies [37].

### Statistical analysis

Data are presented as mean ± SEM for all data. For multiple comparisons, one-way ANOVA followed by Tukey’s test was used to compare between each group. Long-rank method and Gehan–Breslow–Wilcoxon method were used to compare survival curves. All the tests were performed GraphPad Prism version 6. All assays for drug effects were done by double-blinded experiments. For all experiments *p* values < 0.05 were considered significant.

## Results

### Febuxostat (FBX) ameliorates age-dependent damage to body wall muscle cells in *Caenorhabditis elegans*

**Fig. 1:**
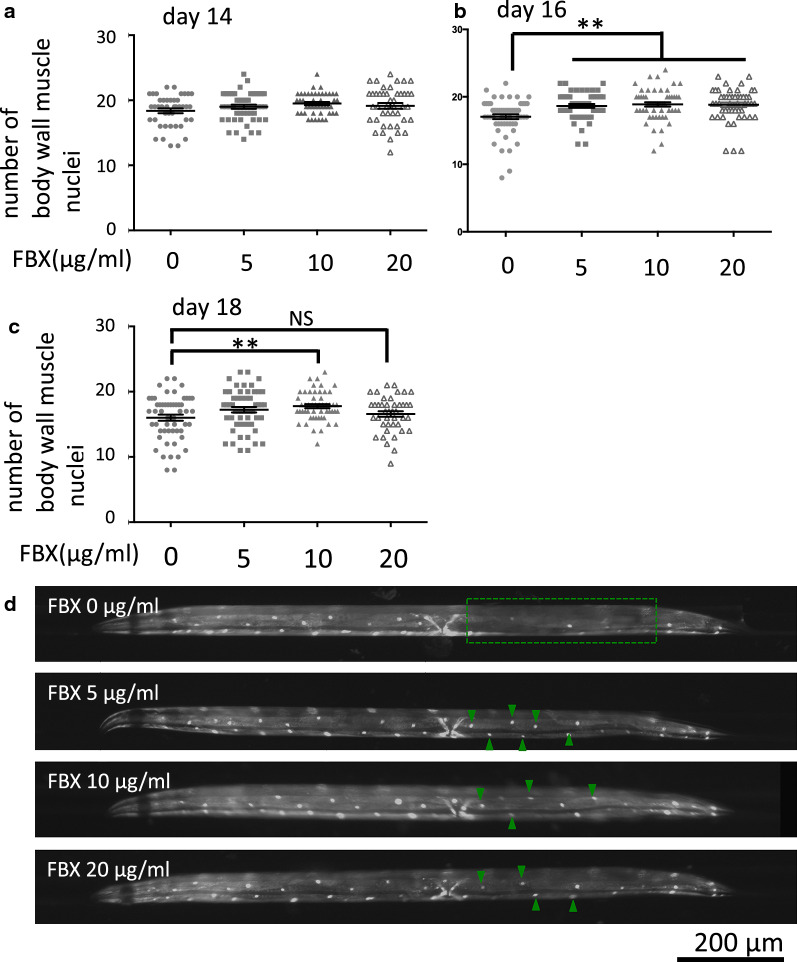
FBX has a protective effect on body wall muscle cells. **A**–**C** Wild-type animals were cultured on a medium containing FBX at the concentration indicated on abscissae, and the numbers of body wall muscle cell nuclei in one bundle per animal were counted on Days 14 (**A**), 16 (**B**), and 18 (**C**). **P < 0.005. **A** Number of trials: three times for each condition. Total number of FBX (0, 5, 10, and 20 µg/ml)-treated animals: 43, 48, 45, and 44, respectively. Mean value of FBX (0, 5, 10, and 20 µg/ml)-treated animals: 18.37 (SEM, ± 0.37), 19.00 (SEM, ± 0.33), 19.49 (SEM, ± 0.25), and 19.14 (SEM, ± 0.43), respectively. **B** Number of trials: three times for each condition. Total number of FBX (0, 5, 10, and 20 µg/ml)-treated animals: 55, 52, 52, and 50, respectively. Mean value of FBX (0, 5, 10, and 20 µg/ml)-treated animals: 17.05 (SEM, ± 0.36), 18.65 (SEM, ± 0.28), 18.88 (SEM, ± 0.33), and 18.82 (SEM, ± 0.34), respectively. **C** Number of trials: three times for each condition. Total number of FBX (0, 5, 10, and 20 µg/ml)-treated animals: 54, 54, 52, and 42, respectively. NS, not significant. Mean value of FBX (0, 5, 10, and 20 µg/ml)-treated animals: 16.02 (SEM, ± 0.47), 17.24 (SEM, ± 0.42), 17.79 (SEM, ± 0.31), and 16.60 (SEM, ± 0.42), respectively. **D** Representative images of the body wall muscle nuclei at Day 16. Two bundles of nuclei are seen in the focal plane. The number of visible nuclei is decreased in the area encircled by the dotted line at the FBX-free condition. Arrowheads indicate nuclei visible in FBX-treated animals but not visible in FBX-free condition

FBX, an inhibitor of XO/XDH, has been shown to increase intracellular adenosine triphosphate (ATP) [22, 38]. In addition, energy loss due to mitochondrial dysfunction or impairment is a mechanism of aging [18, 19]. In particular, muscle aging is correlated with increased mortality and an increased risk of developing an aging-related disease [39, 40]. The efficacy of FBX was tested in *C. elegans,* and the effects of FBX on muscle were examined. Evidence for age-related muscle deterioration was monitored by green fluorescent protein (GFP)-tagged proteins localized to the body wall muscle nuclei (*Pmyo-3::GFP*/nuclear localization signal [NLS]). *Pmyo-3::GFP/NLS*-expressing *ccIs4251* were grown on FBX-supplemented medium, and the number of detectable GFP-labeled body wall muscle nuclei was counted on Days 14, 16, and 18. On Day 14, FBX had no effect (Fig. 1A, mean value of FBX (0, 5, 10, and 20 µg/ml)-treated animals: 18.37 (SEM, ± 0.37), 19.00 (SEM, ± 0.33), 19.49 (SEM, ± 0.25), and 19.14 (SEM, ± 0.43), respectively). On Day 16, the numbers of detectable GFP-labeled body wall muscle nuclei were approximately 10% higher in *C. elegans* reared on a medium supplemented with FBX at 5, 10, and 20 µg/ml (Fig. 1B, D, mean value of FBX (0, 5, 10, and 20 µg/ml)-treated animals: 17.05 (SEM, ± 0.36), 18.65 (SEM, ± 0.28), 18.88 (SEM, ± 0.33), and 18.82 (SEM, ± 0.34), respectively). On Day 18, more detectable GFP-labeled nuclei of the body wall muscle were found in *C. elegans* reared on a medium supplemented with FBX at 10 µg/ml (approximately 7% higher) than in those reared without FBX. FBX at 20 µg/ml showed not significantly (Fig. 1C, mean value of FBX (0, 5, 10, and 20 µg/ml)-treated animals: 16.02 (SEM, ± 0.47), 17.24 (SEM, ± 0.42), 17.79 (SEM, ± 0.31), and 16.60 (SEM, ± 0.42), respectively.

Mitochondrial defects, such as increased fragmentation and reduced mitochondrial volume, are said to be observed in the body wall muscles of aged *C. elegans*. In aged *C. elegans*, neuronal mitochondria exhibit ultrastructural changes, including loss of visible membrane and cristae structures [41]. We observed mitochondria in the body wall muscle cells of aged *C. elegans* by transmission electron microscopy (Additional file 1: Fig. S1). Broken mitochondrial cristae and membrane structures were observed in the animals not treated with FBX. In contrast, the mitochondrial cristae and membrane structures were intact in the FBX-treated worms (Additional file 1: Fig. S1). This finding indicates that the addition of FBX protects against age-related mitochondrial deterioration.

Furthermore, a strain expressing mitochondrial matrix-targeted GFP (mitoGFP) under the control of the *myo-3* promoter (*Pmyo-3::mitoGFP*) [37] was used to monitor the mitochondrial morphology in aging body wall muscle cells. A qualitative analysis of the changes to mitochondrial morphology with age was performed. There was no clear effect of FBX on mitochondrial fragmentation (Additional file 1: Fig. S2A–C).

### FBX increases resistance to the mitochondrial inhibitor NaN_3_

**Fig. 2:**
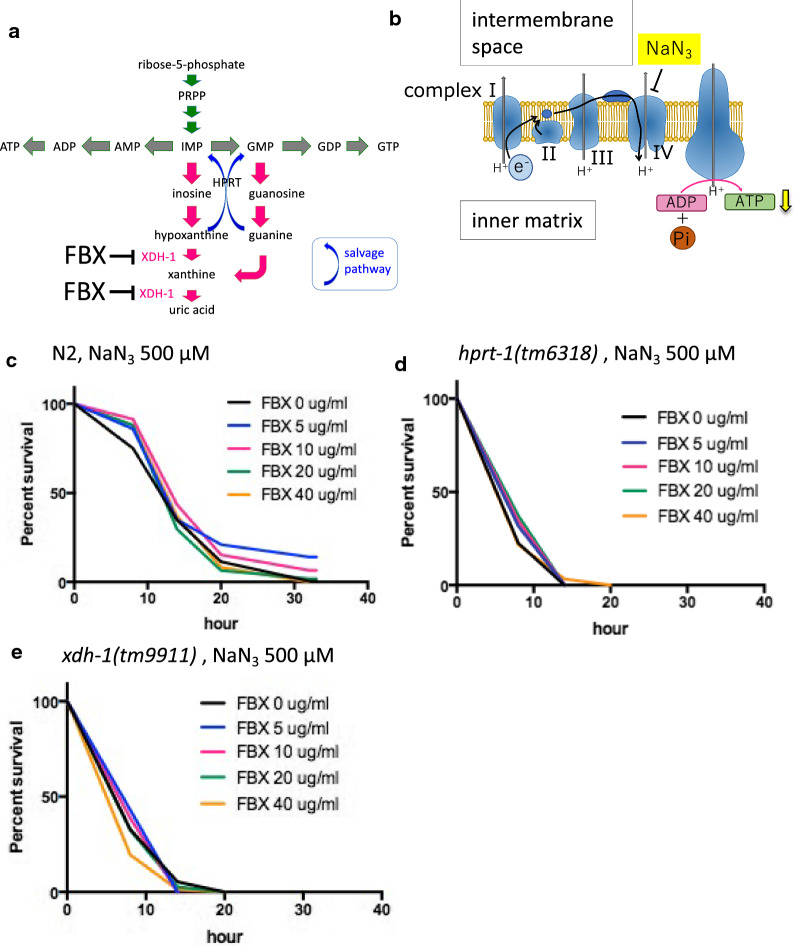
FBX treatment confers resistance to mitochondrial inhibitors in N2 worms. **A** Metabolic pathways of purine nucleotides and active sites of FBX. **B** Schematic diagram of the mitochondrial electron transport chain and the active site of a mitochondrial inhibitor, NaN_3_. **C** The mitochondrial inhibitor NaN_3_ (500 µM) and FBX (0, 5, 10, 20, and 40 µg/ml) were added to wild-type nematodes (N2) at the L4 stage, and the survival time was measured. Log-rank *P* value: FBX 0 µg/ml vs FBX 5 µg/ml = 0.0117. Log-rank *P* value: FBX 0 µg/ml vs FBX 10 µg/ml = 0.0406. Survival rates of 37% at FBX (0, 5, 10, 20 and 40 µg/ml)-treated animals: 13.68, 13.77, 15.38, 13.24, and 14.01 h, respectively. Number of trials: FBX 0 µg/ml, 8 times; FBX 5 µg/ml, 3 times; FBX 10 µg/ml, 3 times; FBX 20 µg/ml, 4 times; FBX 40 µg/ml, 4 times. Total number of FBX (0, 5, 10, 20, and 40 µg/ml)-treated animals: 200, 49, 43, 106, and 108, respectively. NS, not significant. **D** NaN_3_ (500 µM) and FBX (0, 5, 10, 20, and 40 µg/ml) were added to the *hprt-1* mutant (*tm6318*) nematodes in the L4 stage, and survival times were measured. Log-rank *P* value: not significant. Survival rates of 37% at FBX (0, 5, 10, 20, and 40 µg/ml)-treated animals: day 6.71, 7.38, 7.70, 8.04 and 6.43, respectively. Number of trials: FBX 0 µg/ml, 4 times; FBX 5 µg/ml, 3 times; FBX 10 µg/ml, 3 times; FBX 20 µg/ml, 3 times; FBX 40 µg/ml, 3 times. Total number of FBX (0, 5, 10, 20, and 40 µg/ml)-treated animals: 102, 60, 55, 59, and 60, respectively. **E** NaN_3_ (500 µM) and FBX (0, 5, 10, 20, and 40 µg/ml) were added to the L4 stage of the *xdh-1* mutant (*tm9911*) nematodes, and survival time was measured. Log-rank *P* value: not significant. Survival rates of 37% at FBX (0, 5, 10, 20, and 40 µg/ml)-treated animals: 7.51, 8.85, 8.23, 7.46, and 6.26 h, respectively. Number of trials: FBX 0 µg/ml, 10 times; FBX 5 µg/ml, 3 times; FBX 10 µg/ml, 4 times; FBX 20 µg/ml, 3 times; FBX 40 µg/ml, 4 times. Total number of FBX (0, 5, 10, 20, and 40 µg/ml)-treated animals: 225, 79, 80, 74, and 77, respectively.

Because FBX effectively protected muscle cells in *C. elegans*, we considered that FBX could also inhibit the XDH-1 activity in *C. elegans*. FBX is known to be an inhibitor of XO/XDH in humans (Fig. 2A). The amount of uric acid (UA) in the body of worms treated with FBX was measured to investigate the effect of FBX on *C. elegans* XDH-1, which metabolizes xanthine to give rise to UA (Additional file 1: Fig. S3). FBX at concentrations of 5 and 10 µg/ml did not change the amount of UA in *C. elegans*, but 20 µg/ml FBX decreased the amount of UA by approximately 50% (Additional file 1: Fig. S3). This finding indicates that FBX acts as an inhibitor of XDH-1 in *C. elegans*, as it does in humans. Since 5 and 10 µg/ml FBX had effects on muscle cell survival above, we speculated that the unchanged UA concentration was caused by the kinetics of UA excretion and not the inhibition of XDH-1. The protective effect of FBX on the mitochondria (Additional file 1: Fig. S1) was hypothesized since FBX increased undegraded hypoxanthine, increased the salvage pathway of nucleic acid metabolism, and increased ATP synthesis (Fig. 2A). To test this hypothesis, NaN_3_ was administered to wild-type *C. elegans* to inhibit mitochondrial function, and the effects of FBX were observed. It is known that NaN_3_ [42] inhibits oxidative phosphorylation via inhibition of Complex IV/cytochrome c oxidase, the final enzyme in the mitochondrial electron transport chain, thereby resulting in a depletion of intracellular ATP (Fig. 2B). When NaN_3_ was added at 500 µM, all wild-type animals died after 32 h. When FBX (5 and 10 µg/ml) was added with NaN_3_ to wild-type animals, the survival rate of the worms increased. However, the addition of FBX at 20 and 40 µg/ml did not increase the survival rate (Fig. 2C). In the mutant lacking the enzyme that acts in the salvage pathway (*hprt-1*), FBX did not restore the survival rate upon NaN_3_ addition (Fig. 2D, Additional file 1: Fig. S4A). The same experiment was performed using *xdh-1* mutants (*tm9919* and *tm9909*) (Fig. 2E, Additional file 1: Fig. S4B). Unexpectedly, *xdh-1* mutant viability was reduced by 500 µM NaN_3_ treatment compared to wild-type animals. (Median survival times of FBX (0, 5, 10, 20, and 40 µg/ml)-treated *xdh-1(tm9911)* mutant animals: 8, 8, 8, 8, and 8 h, respectively.) Median survival times of FBX (0, 5, 10, 20, and 40 µg/ml)-treated wild-type animals: 14, 14, 14, 14, and 14 h, respectively (Fig. 2C, E). The same tendency was observed on *xdh-1(tm9909)* mutant animals or when NaN_3_ was added at 400 µM (Additional file 1: Figs. S5A–C, S6A, B). The survival rate of *xdh-1* mutant (*ok3234*) that lacks the NAD-binding domain but restores the xanthine dehydrogenase domain was similar to that of the wild-type by treated with NaN_3_ (Additional file 1: Fig. S6C, D).

**Fig. 3:**
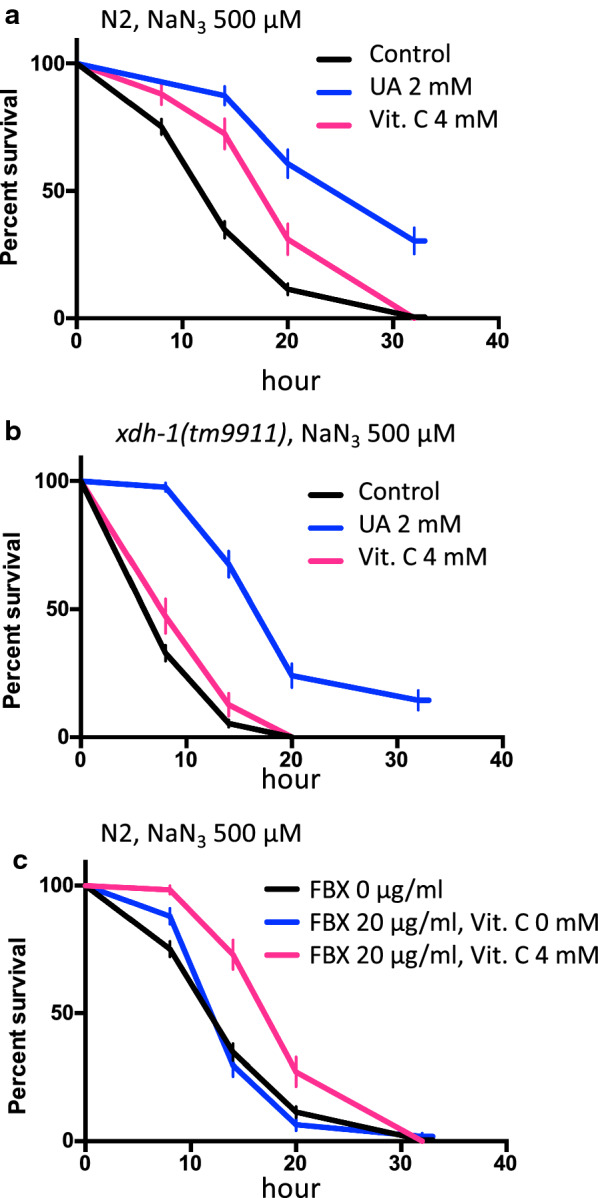
The combined use of uric acid and sodium ascorbate increases the tolerance of *C. elegans* to a mitochondrial inhibitor. **A** NaN_3_ (500 µM) and uric acid (UA, 0, 2 mM) or sodium ascorbate (Vit. C, 0, 4 mM) were supplemented to wild-type (N2) animals at the L4 stage, and the survival time was measured. Log-rank *P* value: Control vs UA 2 mM < 0.0001. Log-rank *P* value: Control vs Vit. C 4 mM < 0.0001. Number of trials: Control, 8 times; UA 2 mM, 3 times; Vit. C 4 mM, 3 times. Total number of Control, UA 2 mM, and Vit. C 4 mM-treated animals: 200, 55, and 58, respectively. **B** NaN_3_ (500 µM) and uric acid (0, 2 mM) or sodium ascorbate (0, 4 mM) were supplemented with the *xdh-1* mutant (*tm9911*) animals at the L4 stage, and the survival time was measured. Log-rank *P* value: Control vs UA 2 mM < 0.0001. Log-rank *P* value: Control vs Vit. C 4 mM = 0.0204. Number of trials: Control, 10 times; UA 2 mM, 3 times; Vit. C 4 mM, 3 times. Total number of Control, UA 2 mM, and Vit. C 4 mM-treated animals: 225, 71, and 55, respectively. **C** NaN_3_ (500 µM), FBX (0, 20 µg/ml), and sodium ascorbate (0, 4 mM) were administered to wild-type (N2) animals in the L4 stage, and the survival time was measured. Log-rank *P* value: FBX 0 µg/ml vs. FBX 20 µg/ml, Vit C 4 mM < 0.0001. Log-rank *P* value: FBX 20 µg/ml vs. FBX 20 µg/ml, Vit C 4 mM < 0.0001. Number of trials: Control, 8 times; UA 2 mM, 3 times; FBX 20 µg/ml + Vit. C 4 mM, 3 times. Total number of FBX 0 µg/ml, FBX 20 µg/ml, and FBX 20 µg/ml + Vit. C 4 mM-treated animals: 200, 106, and 59, respectively

**Fig. 4:**
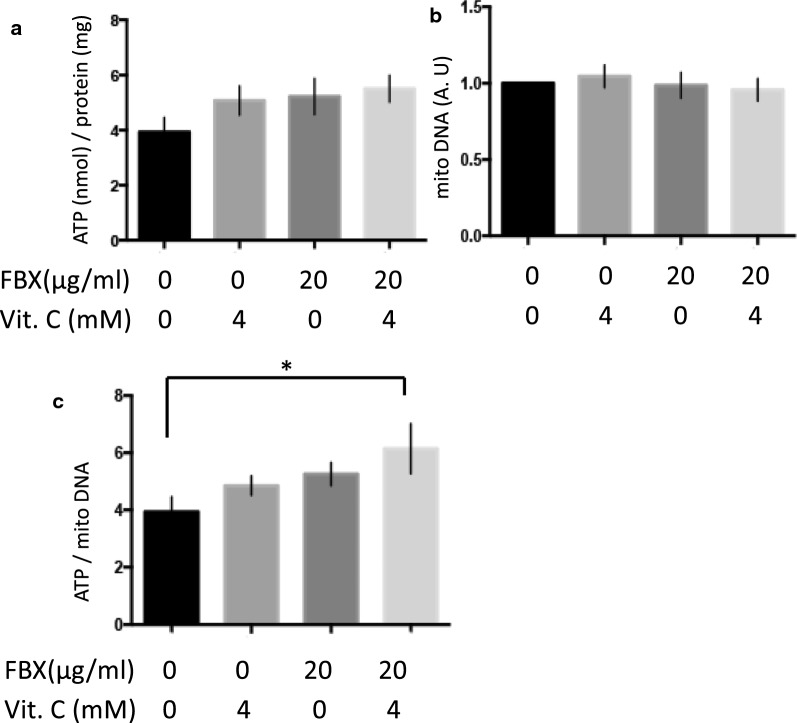
ATP levels in wild-type (N2) animals are increased by FBX and vitamin C in the presence of a mitochondrial inhibitor *N* = 5. The amount of ATP (**A)** and the amounts of mitochondrial DNA (**B)** were determined by adding FBX (0 and 20 µg/ml) and sodium ascorbate (Vit. C, 0 and 4 mM) to N2 at the L4 stage. The amount of ATP was normalized against the protein concentration. **C** The amount of ATP was normalized against mitochondrial DNA content. *P = 0.0471

The survival rate of worms with *xdh-1* mutations (*tm9911* and *tm9909*) was lower than that of the wild-type worms when NaN_3_ was added (Fig. 2E, median survival times of FBX (0, 5, 10, 20, and 40 µg/ml)-treated wild-type animals: all of 14 h. Median survival times of FBX (0, 5, 10, 20, and 40 µg/ml)-treated *tm9911*: all of 8 h) (Additional file 1: Figs. S5C, S6A, B). Because deletions in the *xdh-1* alleles (*tm9911* and *tm9909*) almost completely span the whole gene, the mutants are likely null mutants (Additional file 1: Fig. S4). This suggests that the supplementation of NaN_3_ shortens the survival time of *xdh-1* mutant animals (*tm9911* and *tm9909*) compared to the wild-type animals because *xdh-1* mutant animals have almost no UA (Additional file 1: Fig. S3). To test this possibility, 2 mM UA was added to the medium, and the viability of wild-type and *xdh*-1 mutant animals (*tm9911* and *tm9909*) upon the addition of NaN_3_ was examined. UA increased the resistance to NaN_3_ in both wild-type and *xdh-1* mutant animals (*tm9911* and *tm9909*) (Fig. 3A, B, (A) median survival times of Control, UA 2 mM, and Vit. C 4 mM-treated wild-type animals: 14, 32, and 20 h, respectively. (B) Median survival times of Control, UA 2 mM, and Vit. C 4 mM-treated *tm9911*: 8, 20, and 8 h, respectively.) (Additional file 1: Fig. S7A). Since UA is known to be an antioxidant that exists in vivo [10], another antioxidant (sodium ascorbate, vitamin C) was added, and resistance to NaN_3_ was examined. In wild-type animals, resistance to NaN_3_ increased when vitamin C was added. This was similar to the effects of UA, although the effects of vitamin C were smaller (Fig. 3A). The *xdh-1* mutant (*tm9911*) and *hprt-1* mutant (*tm6318*) animals showed no changes in resistance to NaN_3_ upon vitamin C treatment (Fig. 3B, Additional file 1: Fig. S7B). This indicates that UA acts as an antioxidant, and to some extent, this action can be substituted by vitamin C. When FBX was added to wild-type animals at 20 µg/ml, resistance to NaN_3_ was not observed (Figs. 2C, 3C), and when 4 mM vitamin C was added together with FBX, resistance to NaN_3_ increased (Fig. 3C, median survival times of FBX 0 µg/ml, FBX 20 µg/ml + Vit. C 0 mM, and FBX 20 µg/ml + Vit. C 4 mM-treated wild-type animals: 14, 14, and 20 h, respectively). The increased resistance to NaN_3_ by FBX appears to be due to the activation of the salvage pathway and the increased ATP. We measured the amount of ATP in wild-type animals when FBX and/or vitamin C were added. The amount of ATP per mitochondria increased when both FBX and vitamin C were added (Fig. 4A–C).

### The addition of FBX and vitamin C extends the lifespan of *C. elegans*

**Fig. 5:**
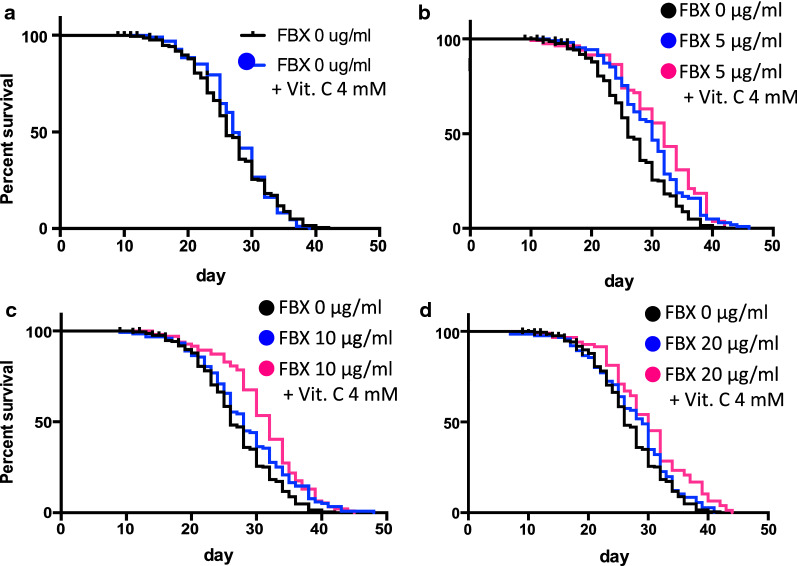
The combination of FBX and sodium ascorbate prolongs the lifespan of wild-type (N2) animals. **A**–**D** Lifespan of N2 worms treated with different concentrations of FBX (0, 5, 10, and 20 µg/ml) and sodium ascorbate (Vit. C, 0 and 4 mM). **A** Log-rank *P* value: FBX 0 µg/ml vs FBX 0 µg/ml + Vit. C 4 mM = not significant. **B** Log-rank *P* value: FBX 0 µg/ml vs FBX 5 µg/ml = 0.0003. Log-rank *P* value: FBX 5 µg/ml vs FBX 5  µg /ml + Vit. C 4 mM = 0.1232. Log-rank *P* value: FBX 0 µg/ml vs FBX 5 µg/ml + Vit. C 4 mM < 0.0001. **C** Log-rank *P* value: FBX 0 µg/ml vs FBX 10 µg/ml = 0.0067. Log-rank *P* value: FBX 10 µg/ml vs FBX 10 µg/ml + Vit. C 4 mM = 0.052. Log-rank *P* value: FBX 0 µg/ml vs FBX 10 µg/ml + Vit. C 4 mM < 0.0001. **D** Log-rank *P* value: FBX 0 µg/ml vs FBX 20 µg/ml = 0.1442. Log-rank *P* value: FBX 20 µg/ml vs FBX 20 µg/ml + Vit. C 4 mM = 0.0129. Log-rank *P* value: FBX 0 µg/ml vs FBX 20 µg/ml + Vit. C 4 mM < 0.0001. Detailed *P* values are shown in Table 1. Median values, maximum lifespan, number of trials, and total number of animals are shown in Table 2

Treatment of wild-type *C. elegans* with UA, an antioxidant, is known to prolong its lifespan [11]. Energy loss due to mitochondrial dysfunction or impairment is suggested to be a mechanism of aging [18, 19]. We have shown that the mitochondria were protected by supplementation with FBX. Therefore, FBX and vitamin C were added to wild-type animals, and the lifespan was measured. Treatment with vitamin C alone did not improve the lifespan (Fig. 5A, Tables 1, 2). FBX at 5 µg/ml alone had an effect on lifespan extension compared to FBX-free conditions (Fig. 5B, Tables 1, 2). The lifespan when FBX was administered at 10 µg/ml was similar or slightly shorter as the lifespan when FBX was administered at 5 µg/ml. (Fig. 5C, Tables 1, 2). FBX at 20 µg/ml alone did not differ significantly from FBX-free conditions (Fig. 5D, Tables 1, 2). In addition, the coadministration of FBX and 4 mM vitamin C prolonged the lifespan of the animals compared to FBX alone (Fig. 5B–D, Tables 1, 2), and the effect was not attenuated even when the FBX concentration was increased to 20 µg/ml (Fig. 5D, Tables 1, 2).

**Table 1:** Curve comparison of lifespan, related to Fig. 5

	Log-rank *P* value	Gehan–Breslow–Wilcoxon *P* value
FBX 0 µg /ml vs FBX 0 µg /ml + VitC 4 mM	0.7856	0.2675
FBX 0 µg /ml vs FBX 5 µg /ml	0.0003	0.0005
FBX 0 µg /ml vs FBX 5 µg /ml + VitC 4 mM	< 0.0001	< 0.0001
FBX 5 µg /ml vs FBX 5 µg /ml + VitC 4 mM	0.1232	0.1365
FBX 0 µg /ml vs FBX 10 µg /ml	0.0067	0.0718
FBX 0 µg /ml vs FBX 10 µg /ml + VitC 4 mM	< 0.0001	< 0.0001
FBX 10 µg /ml vs FBX 10 µg /ml + VitC 4 mM	0.052	0.0034
FBX 0 µg /ml vs FBX 20 µg /ml	0.1442	0.2166
FBX 0 µg /ml vs FBX 20 µg /ml + VitC 4 mM	< 0.0001	0.0015
FBX 20 µg /ml vs FBX 20 µg /ml + VitC 4 mM	0.0129	0.0596

**Table 2:** Effect of FBX and sodium ascorbate on lifespan, related to Fig. 5

	Median survival (SEM)	Max. lifespan	Number of trials	Total number of animals
FBX 0 µg /ml	26 (3.50)	42	4	204
FBX 0 µg /ml + VitC 4 mM	27 (5.32)	39	2	88
FBX 5 µg /ml	30 (4.96)	46	3	101
FBX 5 µg /ml + VitC 4 mM	32 (5.49)	42	2	82
FBX 10 µg /ml	28 (4.62)	48	3	116
FBX 10 µg /ml + VitC 4 mM	32 (5.12)	45	2	92
FBX 20 µg /ml	29 (4.83)	41	3	107
FBX 20 µg /ml + VitC 4 mM	30 (5.64)	44	2	78

### Coadministration of FBX and vitamin C suppresses the phenotype of a familial Parkinson’s disease nematode model

**Fig. 6:**
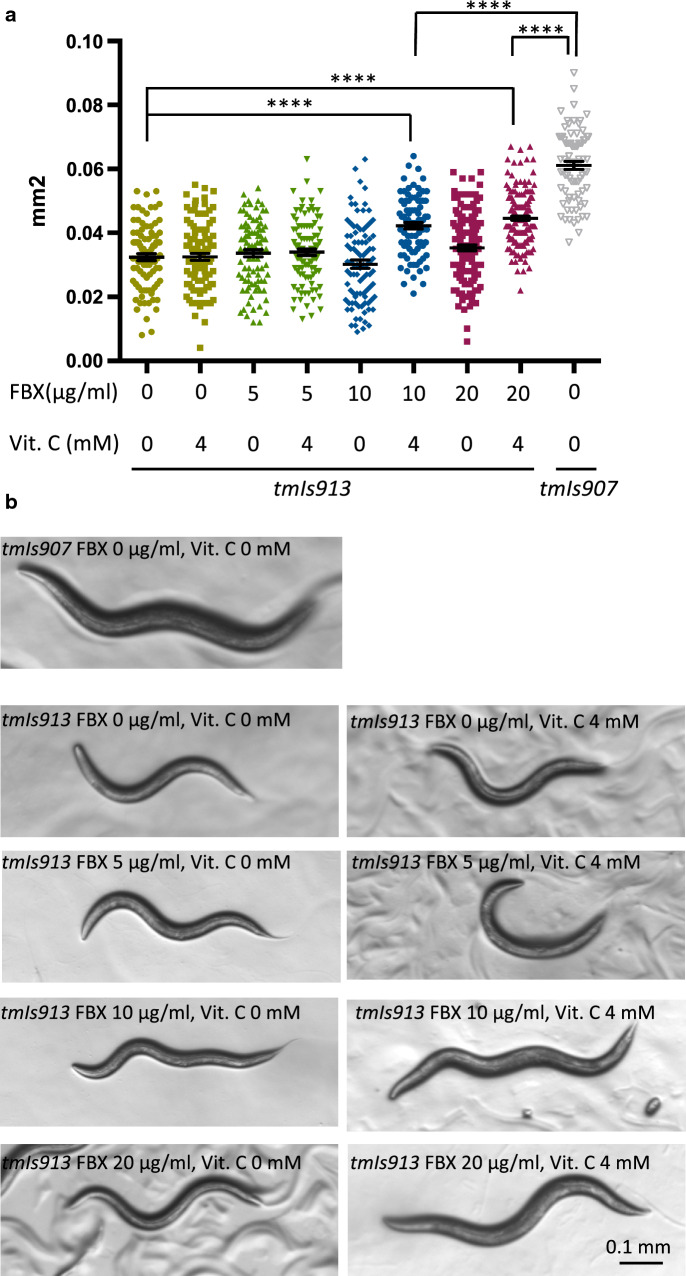
The combined use of FBX and sodium ascorbate treatment suppresses a phenotype of familial Parkinson’s disease in *C. elegans*. **A** FBX (0, 5, 10, and 20 µg/ml) and sodium ascorbate (Vit. C, 0, 4 mM) were added to Tg animals expressing α-synuclein (S129A) (*tmIs913 [Punc-51::α-SynS129A]*) and *tmIs907 [Punc-51::EGFP]* as a control. The body size was quantified at 96 h after bleaching. ****P < 0.0001. Number of trials and total number of animals are shown in Table 3. **B** Representative images of the body size

Treatment of wild-type *C. elegans* with FBX increased number of detectable muscle nuclei (Fig. 1A–D). The degree of nuclear change was reported to correlate with locomotor performance [43]. FBX (0, 5, 10, 20 µg/ml) and vitamin C (0, 4 mM) were administered to transgenic animals expressing α-Syn (S129A) (*tmIs913 [Punc-51::α-SynS129A, Punc-51::EGFP]*) and *tmIs907 [Punc-51::EGFP]* as a control, and the body size was measured 96 h after bleaching. S129A-expressing nematodes showed growth retardation. Administration of vitamin C alone had no growth-promoting effect. The growth-promoting effect of FBX alone was not observed either. In addition, growth was promoted in the presence of FBX (10 and 20 µg/ml) and vitamin C (4 mM) (Fig. 6A, B). These results indicate that the synergistic effect of FBX and vitamin C reduces the toxicity of α-synuclein.

**Table 3:** Number of trials and total number of animals, related to Fig. 6

Strain	Condition	Number of trials	Total number of animals
tmIs913	FBX 0 μg/ml, Vit.c 0 mM	3	88
tmIs913	FBX 0 μg/ml, Vit.c 4 mM	3	98
tmIs913	FBX 5 μg/ml, Vit.c 0 mM	3	89
tmIs913	FBX 5 μg/ml, Vit.c 4 mM	3	97
tmIs913	FBX 10 μg/ml, Vit.c 0 mM	3	91
tmIs913	FBX 10 μg/ml, Vit.c 4 mM	3	87
tmIs913	FBX 20 μg/ml, Vit.c 0 mM	4	142
tmIs913	FBX 20 μg/ml, Vit.c 4 mM	4	166
tmIs907	FBX 0 μg/ml, Vit.c 0 mM	3	80

### FBX administration suppresses the phenotype of a tauopathy model

XO/XDH inhibitors have been reported to suppress the onset of dementia [24, 44]. The effect of FBX was tested using transgenic animals expressing Tau (WT4R) in the nerve (*tmIs390 [Punc-119::WT4R, Pges-1::EGFP]*) as a model for Alzheimer’s disease, which is said to account for approximately 70% of dementia. *tmIs390* has been reported to produce the phenotype of Unc [34]. *tmIs388 [Pges-1::EGFP]* was used as a control, and *tmIs390* was supplemented with FBX (0, 5, 10, and 20 µg/ml). The area of nematode movement over 30 min was examined at 11 days after bleaching. The migration distance of the nematodes was prolonged in the presence of FBX (5 and 10 µg/ml) and was more prolonged in FBX (20 µg/ml) (Additional file 1: Fig. S8).

## Discussion

In this study, we investigated the effects of FBX in *C. elegans*. Low concentrations of FBX elicited protective effects on age-related muscle deterioration (Fig. 1B–D). This effect was not observed on day 14 and was observed after day 16. In young worms, muscles are repaired by the native system of the body. However, in older worms, the muscle repair mechanism declines, which is the reason why the effect of FBX was observed. Furthermore, low concentrations of FBX led to NaN_3_ resistance and longer survival. However, these effects were hardly observed when FBX was added at a high concentration to wild-type animals (Figs. 1C, 2C). And these effects were not observed when at any concentrations of FBX was added to *xdh-1* mutant animals (*tm9911*) (Fig. 2E, Additional file 1: Fig. S5C). When FBX was added to wild-type animals at a high concentration (20 µg/ml), the concentration of UA in the body was reduced by approximately half, and the *xdh-1* mutant animals (*tm9911*) had almost no UA in the body (Additional file 1: Fig. S3). The reason for the decreased drug efficacy of FBX at higher concentrations appears to be caused by reduced UA, an antioxidant. In support of this hypothesis, the addition of antioxidants with a high concentration of FBX led to NaN_3_ resistance (Additional file 1: Fig. S3) and longer lifespan (Fig. 5B–D, Tables 1, 2).

When *xdh-1* mutant animals (*ok3234*) were treated with NaN_3_ (500 μM) and FBX (10 μg/ml), the resistance to NaN_3_ was increased compared with only NaN_3_ treatment in *xdh-1 (tm9911)* (Fig. 2E, Additional file 1: Fig. S6D). *xdh-1* (*ok3234*) mutant showed the above results due to absence of the NAD-binding site. XDH-1 contains FAD and molybdopterin domains [45]. The FAD domain is the NAD-binding site, and the molybdopterin domain is the redox center. XDH is an enzyme that metabolizes hypoxanthine to xanthine by hydroxylation and metabolizes xanthine to uric acid by hydroxylation downstream of the purine metabolic pathway. The fact that the *xdh-1* mutant animals (*ok3234*) lacking only the NAD-binding site show a normal concentration of ROS [45] also supports this possibility.

In the present study, we showed that FBX protects mitochondria (Additional file 1: Fig. S1). Simultaneous administration of FBX (20 μg/ml) and vitamin C in the presence of NaN_3_ did not decrease ATP production in mitochondria (Fig. 4). Together with the high sensitivity to NaN_3_ of *hprt-1 (tm6318)* mutant animals, which cannot use the salvage pathway, these results indicate that FBX activated the salvage pathway of purine metabolism and contributed to ATP production. Thus, it reduced mitochondrial overuse and led to mitochondrial protection.

We demonstrated that FBX and vitamin C have synergistic effects on reducing α-synuclein toxicity (Fig. 6A, B), and FBX administration represses the tauopathy phenotypes (Additional file 1: Fig. S8A, B). Mitochondrial or bioenergetic dysfunction may be related to the etiology of Alzheimer’s disease (AD) and Parkinson’s disease (PD). Additionally, gout patients treated with UA-lowering drugs have a lower risk of both vascular and nonvascular dementia [44]. Furthermore, impaired removal of defective mitochondria is a pivotal event in AD pathogenesis, suggesting that targeting the maintenance of mitochondrial quality may be a promising therapeutic strategy [46]. Based on the protective effects of FBX on mitochondria in *C. elegans* (Additional file 1: Fig. S1), FBX has a potential therapeutic or preventive agent for the treatment of AD and PD. The therapeutic effects of FBX may be enhanced by coadministration of FBX and vitamin C. A recent study identified FBX as a potential new treatment for AD using artificial intelligence approaches [47].

In humans, vitamin C intake is inversely associated with serum uric acid concentrations [48]. Vitamin C decreases free radical-induced damage to somatic cells and likely modulates serum uric acid concentration via its uricosuric effect [48–50]. Furthermore, vitamin C intake improves renal function and increases the glomerular filtration rate [50–52].

In the previous reports [12, 13, 15–19], there were conflicting reports on the relationship between mitochondrial function, ATP contents and lifespan. Our results showed that it is important to maintain ATP levels for lifespan extension and that protection from ROS generated during ATP production is also important. We showed that FBX administration could increase ATP levels while extending the lifespan. These results indicate the potential that the combination of FBX and vitamin C, antioxidant, would not only increase the therapeutic treatment of gout, but also have a preventive effect on other diseases and prolong lifespan.

## Conclusions

In this paper, we have shown that coadministration of FBX and vitamin C balanced mitochondrial function, ATP levels, and antioxidant levels can prevent aging and extend lifespan. Furthermore, FBX has a potential as therapeutic or preventive agent for the treatment of AD and PD.

### Supplementary Information

**Additional file1**: **Figure S1.** Wild-type animals were cultured on a medium containing FBX (right) or FBX-free condition (left). The mitochondria of 18-day-old *C. elegans* were observed using transmission electron microscopy. In 18-day-old animals, some of cristae were difficult to resolve at FBX-free conditions (left). In 18-day-old animals which were treated with FBX showed prominent internal cristae structure (right). Arrowheads indicate mitochondria. An enlarged image of the area marked by the square is shown in the bottom row. **Figure S2. **(A-C) Transgenic animals expressing mitoGFP in body wall muscle cells (*ccIs4251 [Pmyo-3::mitoGFP]*) were analyzed on different days after bleach synchronization (A = Day 14, B = Day 16, C = Day 18). Qualitative analysis of mitochondrial morphology during aging. Navy, cyan, green, yellow, and orange represent the percentage of animals displaying tubular, intermediate, fragmented, very fragmented, and undetectable mitochondrial morphology, respectively (n > 90 images). **Figure S3. **FBX changes the uric acid content in *C. elegans*. N2 worms were cultured on a medium containing FBX (0, 5, 10, and 20 µg/ml), and the amounts of uric acid present in the body were measured. As a control, uric acid levels were also measured in the *xdh-1* mutant (*tm9911*). * P = 0.0205, **** P < 0.0001, N = 9. **Figure S4. ***C. elegans* deletion mutants. A schematic diagram of the *hprt-1* (A) and *xdh-1* (B) genes and their mutants. **Figure S5. **FBX (0-40 µM) treatment confers resistance to a mitochondrial inhibitor in wild-type *C. elegans* (N2). *hprt-1* and *xdh-1* mutant animals did not show FBX-dependent resistance to a mitochondrial inhibitor, NaN_3_. A. The mitochondrial inhibitor NaN_3_ (400 µM) and FBX (0, 5, 10, 20, and, 40 µg/ml) were added to wild-type nematodes (N2) at the L4 stage, and the survival time was measured. Log-rank P value: FBX 0 µg/ml vs. FBX 5 µg/ml = 0.0241. Log-rank P value: FBX 0 µg/ml vs. FBX 10 µg/ml = 0.6487. Median survival times of FBX (0, 5, 10, 20, and 40 µg/ml)-treated wild-type animals: 14, 20, 14, 20, and 14 hours, respectively). Number of trials: all 3 times. Total number of FBX (0, 5, 10, 20, and 40 µg/ml)-treated animals: 51, 53, 42, 55, and 54, respectively. B. NaN_3_ (400 µM) and FBX (0, 5, 10, 20, and 40 µg/ml) were added to the L4 stage of *hprt-1* mutant (*tm6318*) nematodes, and the survival time was measured. Median survival times of FBX (0, 5, 10, 20, and 40 µg/ml)-treated *tm6318*: 14, 14, 8, 8, and 8 hours, respectively). Number of trials: FBX 0 µg/ml, 6 times; Others, 3 times. Total number of FBX (0, 5, 10, 20, and 40 µg/ml)-treated animals: 112, 60, 59, 61, and 54, respectively. C. NaN_3_ (400 µM) and FBX (0, 5, 10, 20, and 40 µg/ml) were added to the L4 stage of *xdh-1* mutant (*tm9911*) nematodes, and the survival time was measured. Median survival times of FBX (0, 5, 10, 20, and 40 µg/ml)-treated *tm9911*: 14, 14, 14, 14, and 14 hours, respectively). Number of trials: all 3 times. Total number of FBX (0, 5, 10, 20, and 40 µg/ml)-treated animals: 75, 70, 73, 80, and 77, respectively. **Figure S6. ***xdh-1* mutants lose FBX-dependent resistance to mitochondrial inhibitor. A. NaN_3_ (400 µM) and FBX (0, 5, 10, 20, and 40 µg/ml) were added to the L4 stage of the *xdh-1* mutant (*tm9909*) nematodes, and the survival time was measured. Median survival times of FBX (0, 5, 10, 20, and 40 µg/ml)-treated *tm9909*: 14, 8, 8, 8, and 14 hours, respectively). Number of trials: all 3 times. Total number of FBX (0, 5, 10, 20, and 40 µg/ml)-treated animals: 78, 75, 79, 71 and 68, respectively. B. NaN_3_ (500 µM) and FBX (0, 5, 10, 20, and 40 µg/ml) were added to the L4 stage of the *xdh-1* mutant (*tm9909*) nematodes, and the survival time was measured. Median survival times of FBX (0, 5, 10, 20, and 40 µg/ml)-treated *tm9909*: 8, 8, 8, 8, and 8 hours, respectively). Number of trials: FBX 0 µg/ml, 10 times; Others, 3 times. Total number of FBX (0, 5, 10, 20, and 40 µg/ml)-treated animals: 233, 77, 76, 74 and 74, respectively. C. NaN_3_ (400 µM) and FBX (0, 5, 10, 20, and 40 µg/ml) were added to the L4 stage of the *xdh-1* mutant (*ok3234*) nematodes, and the survival time was measured. Median survival times of FBX (0, 5, 10, 20, and 40 µg/ml)-treated *ok3234*: 14, 14, 14, 14, and 14 hours, respectively). Number of trials: all 3 times. Total number of FBX (0, 5, 10, 20, and 40 µg/ml)-treated animals: 58, 54, 51, 59 and 36, respectively. D. NaN_3_ (500 µM) and FBX (0, 5, 10, 20, and 40 µg/ml) were added to the *xdh-1* mutant (*ok3234*) nematodes at the L4 stage, and the survival time was measured. Median survival times of FBX (0, 5, 10, 20, and 40 µg/ml)-treated *ok3234*: 14, 14, 14, 14, and 14 hours, respectively). Number of trials: all 3 times. Total number of FBX (0, 5, 10, 20, and 40 µg/ml)-treated animals: 60, 56, 59, 58 and 58, respectively. **Figure S7. **Uric acid increases the tolerance of the *xdh-1* mutant (*tm9909*) nematodes to a mitochondrial inhibitor. A. NaN_3_ (500 µM) and uric acid (0, 2 mM) were administered to the L4 stage of *xdh-1* mutant (*tm9909*) nematodes, and survival time was measured. Log-rank P value: Control vs. UA 2mM < 0.0001. Median survival times of Control and UA 2 mM-treated animals: 8, and 20, respectively. Number of trials: Control, 10 times; UA 2 mM, 3 times; Total number of Control, 233; Total number of UA 2 mM, 77. B. NaN_3_ (500 µM) and sodium ascorbate (0, 4 mM) were administered to the L4 stage of *hprt-1* mutant (*tm6318*) nematodes, and survival time was measured. Median survival times of Control and Vit. C 4 mM-treated animals: 8, and 8, respectively. Number of trials: Control, 4 times; Vit. C 4 mM, 3 times; Total number of Control, 102; Total number of Vit. C 4 mM, 69. **Figure S8. **FBX alleviated the locomotory behavior of the Tau-expressing *C. elegans* model of Alzheimer’s disease. FBX (0, 5, 10, and 20 µg/ml) was added to Tg animals expressing Tau. After treatment for eleven days, the distance the worms traveled in 30 minutes was quantified. *** P <0.001, **** P <0.0001. Number of trials: all 4 times. Total number of *tmIs388*: 91. Total number of FBX (0, 5, 10, and 20 µg/ml)-treated *tmIs390*: 124, 127, 125, and 110, respectively. Mean value of *tmIs388*: 7.130, Mean value of FBX (0, 5, 10, and 20 µg/ml)-treated *tmIs390*: 2.346, 3.302, 3.268 and 4.127, respectively. B. Representative images of worm tracking.

## Data Availability

The datasets used and analyzed during the current study are available from the corresponding author on reasonable request.

## Abbreviations

FBX: Febuxostat
XO: Xanthine oxidase
XDH: Xanthine dehydrogenase
AD: Alzheimer’s disease
PD: Parkinson’s disease
EGFP: Enhanced green fluorescent protein
UA: Uric acid
TMP: Trimethylpsoralen
UV: Ultraviolet
Tg: Transgenic
