# Supplementary material for: Regulation of aging by balancing mitochondrial function and antioxidant levels
Source: J Physiol Sci. 2022 Nov 15;72:28. doi: 10.1186/s12576-022-00853-1 (PMC10717039; doi:10.1186/s12576-022-00853-1)
Supplement: Supplementary file 1 — Additional file1: Figure S1. Wild-type animals were cultured on a medium containing FBX (right) or FBX-free condition (left). The mitochondria of 18-day-old C. elegans were observed using transmission electron microscopy. In 18-day-old animals, some of cristae were difficult to resolve at FBX-free conditions (left). In 18-day-old animals which were treated with FBX showed prominent internal cristae structure (right). Arrowheads indicate mitochondria. An enlarged image of the area marked by the square is shown in the bottom row. Figure S2. (A-C) Transgenic animals expressing mitoGFP in body wall muscle cells (ccIs4251 [Pmyo-3::mitoGFP]) were analyzed on different days after bleach synchronization (A = Day 14, B = Day 16, C = Day 18). Qualitative analysis of mitochondrial morphology during aging. Navy, cyan, green, yellow, and orange represent the percentage of animals displaying tubular, intermediate, fragmented, very fragmented, and undetectable mitochondrial morphology, respectively (n > 90 images). Figure S3. FBX changes the uric acid content in C. elegans. N2 worms were cultured on a medium containing FBX (0, 5, 10, and 20 µg/ml), and the amounts of uric acid present in the body were measured. As a control, uric acid levels were also measured in the xdh-1 mutant (tm9911). * P = 0.0205, **** P < 0.0001, N = 9. Figure S4. C. elegans deletion mutants. A schematic diagram of the hprt-1 (A) and xdh-1 (B) genes and their mutants. Figure S5. FBX (0-40 µM) treatment confers resistance to a mitochondrial inhibitor in wild-type C. elegans (N2). hprt-1 and xdh-1 mutant animals did not show FBX-dependent resistance to a mitochondrial inhibitor, NaN3. A. The mitochondrial inhibitor NaN3 (400 µM) and FBX (0, 5, 10, 20, and, 40 µg/ml) were added to wild-type nematodes (N2) at the L4 stage, and the survival time was measured. Log-rank P value: FBX 0 µg/ml vs. FBX 5 µg/ml = 0.0241. Log-rank P value: FBX 0 µg/ml vs. FBX 10 µg/ml = 0.6487. Median survival times of FBX (0, 5, 10, 20 [file 12576_2022_853_MOESM1_ESM.pdf]

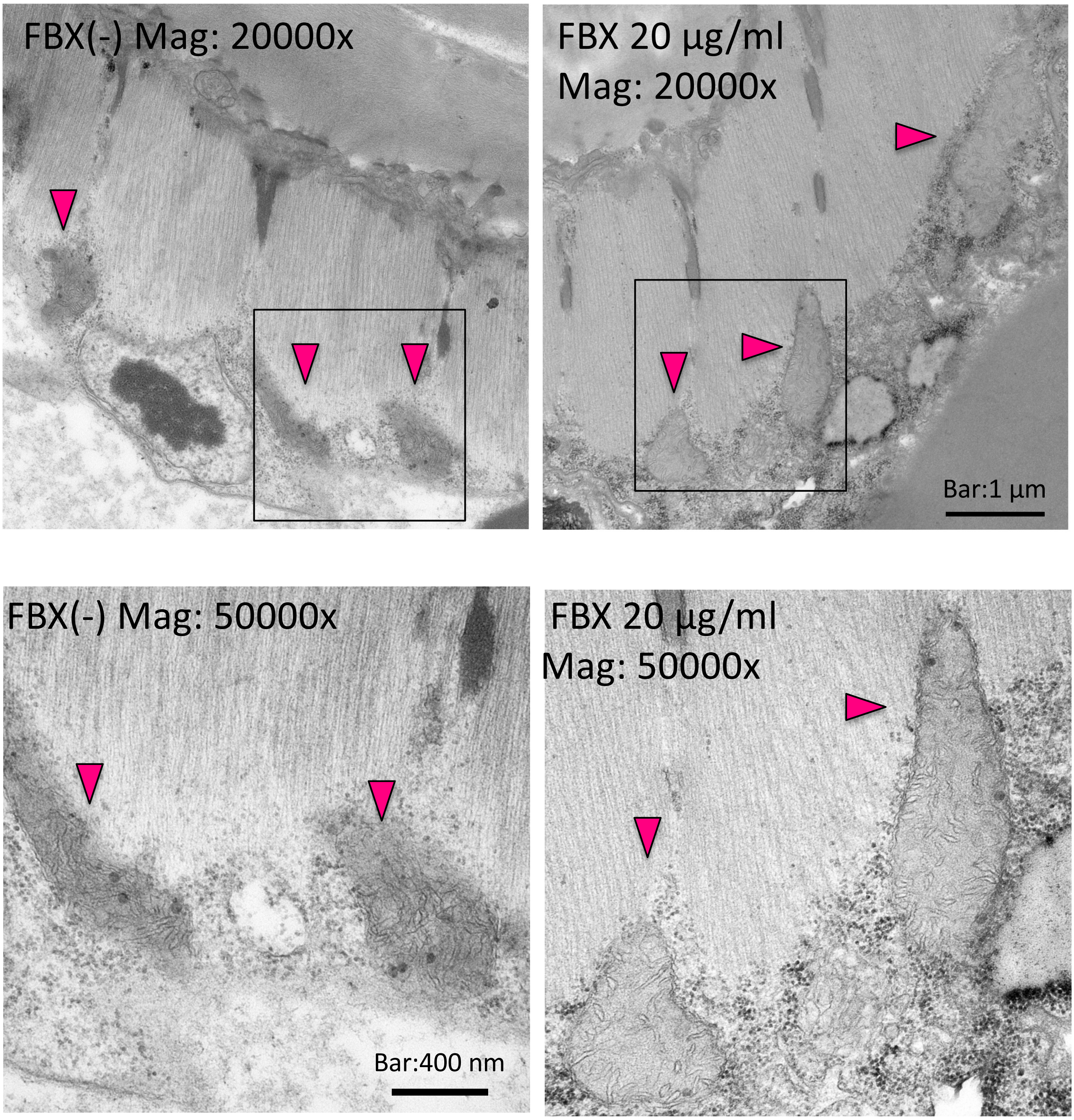

supplementary Fig. 1

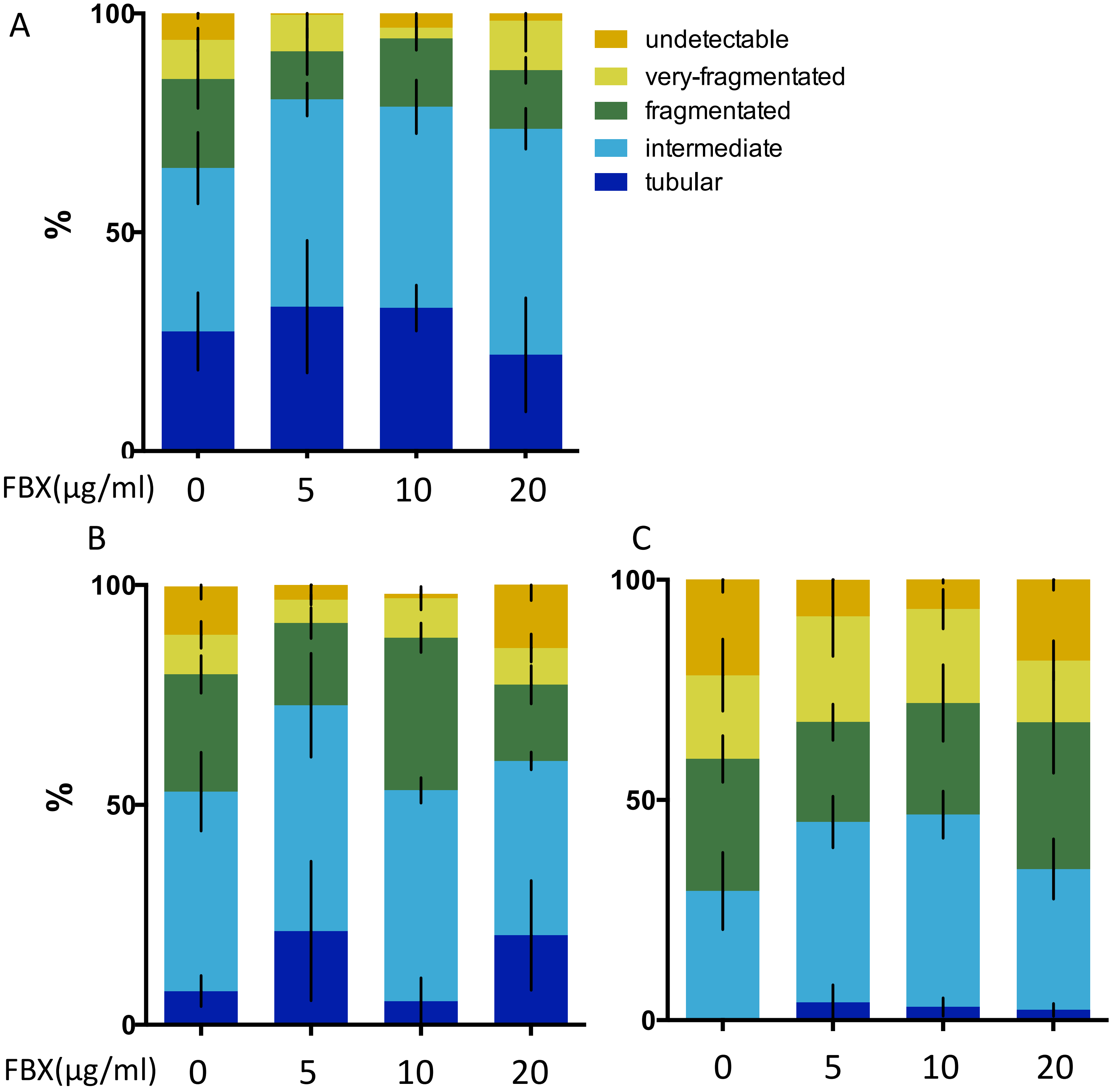

supplementary Fig. 2

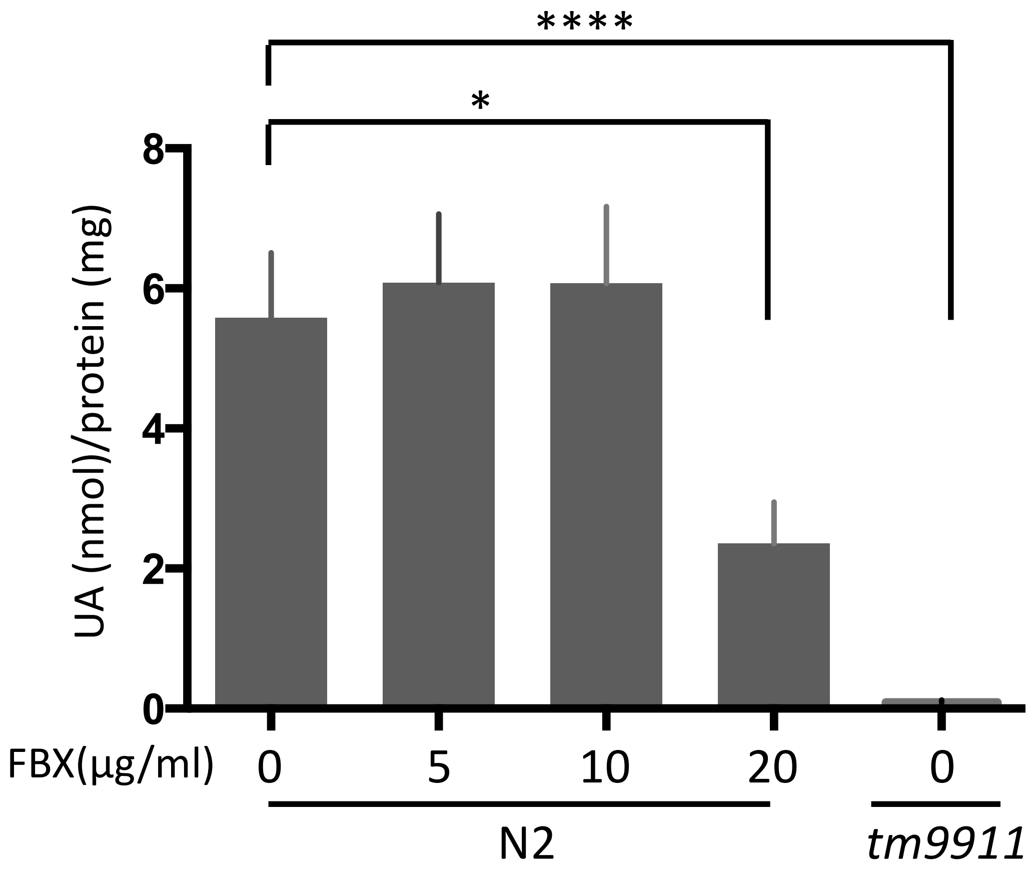

supplementary Fig. 3

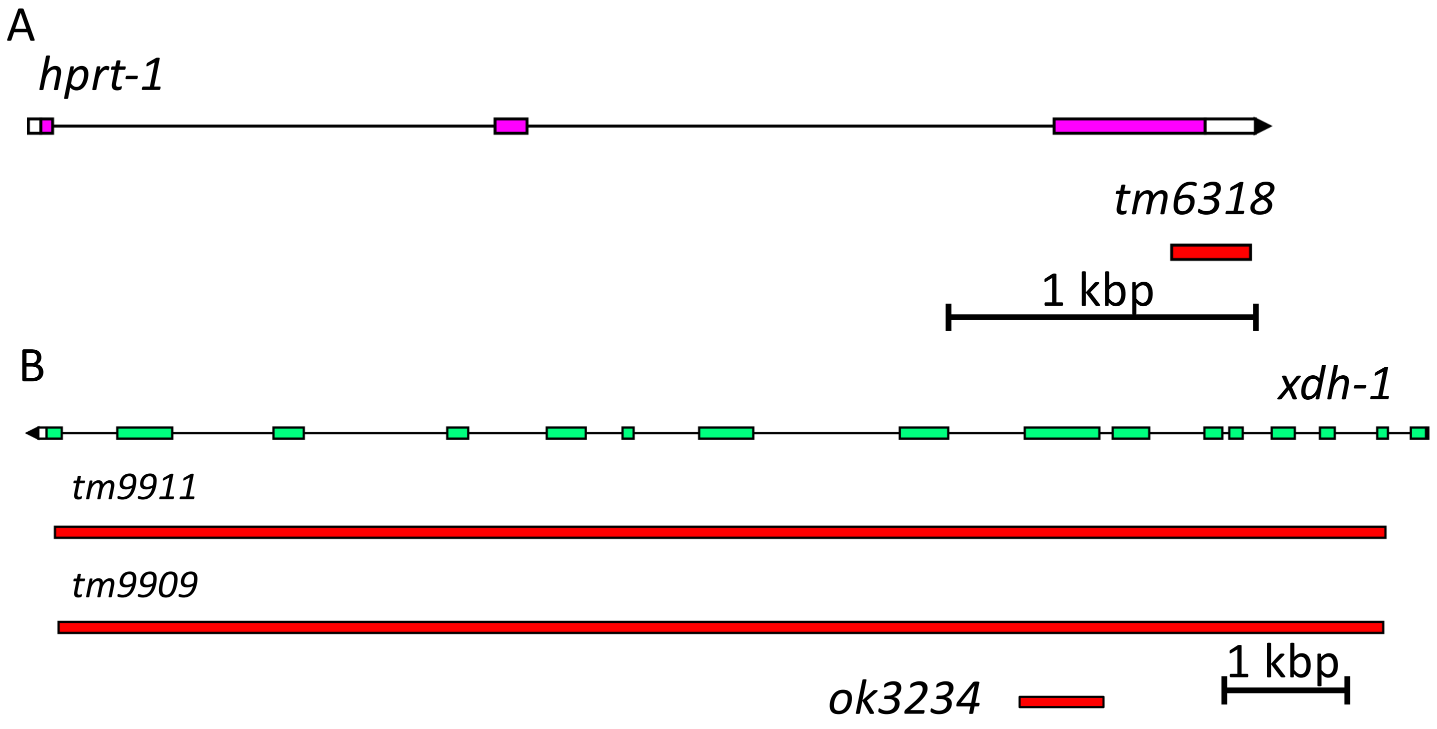

supplementary Fig. 4

A

N2, NaN_3 400 μM

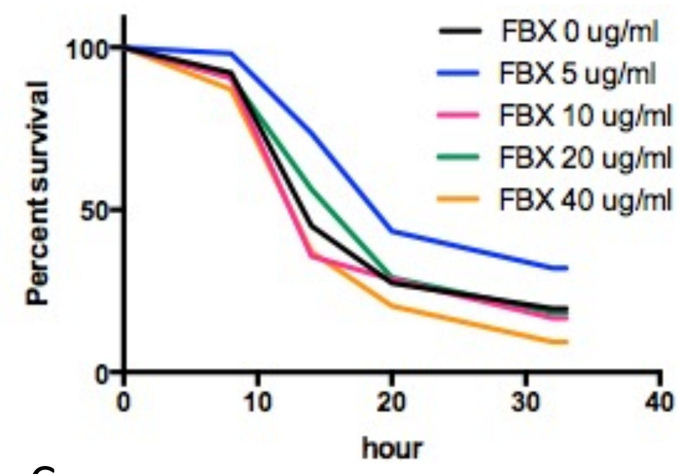

B

hprt-1(tm6318) , NaN_3 400 μM

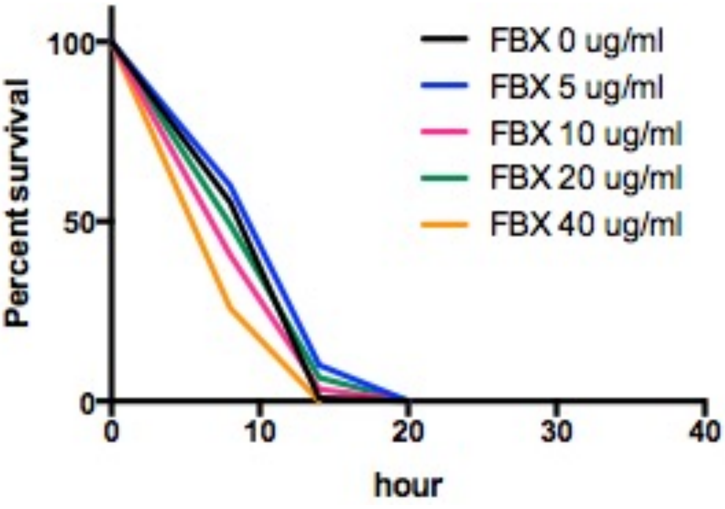

C

xdh-1(tm9911) , NaN_3 400 μM

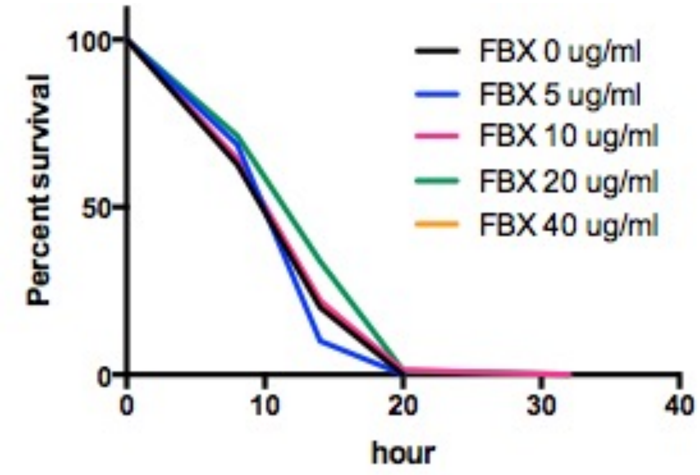

supplementary Fig. 5

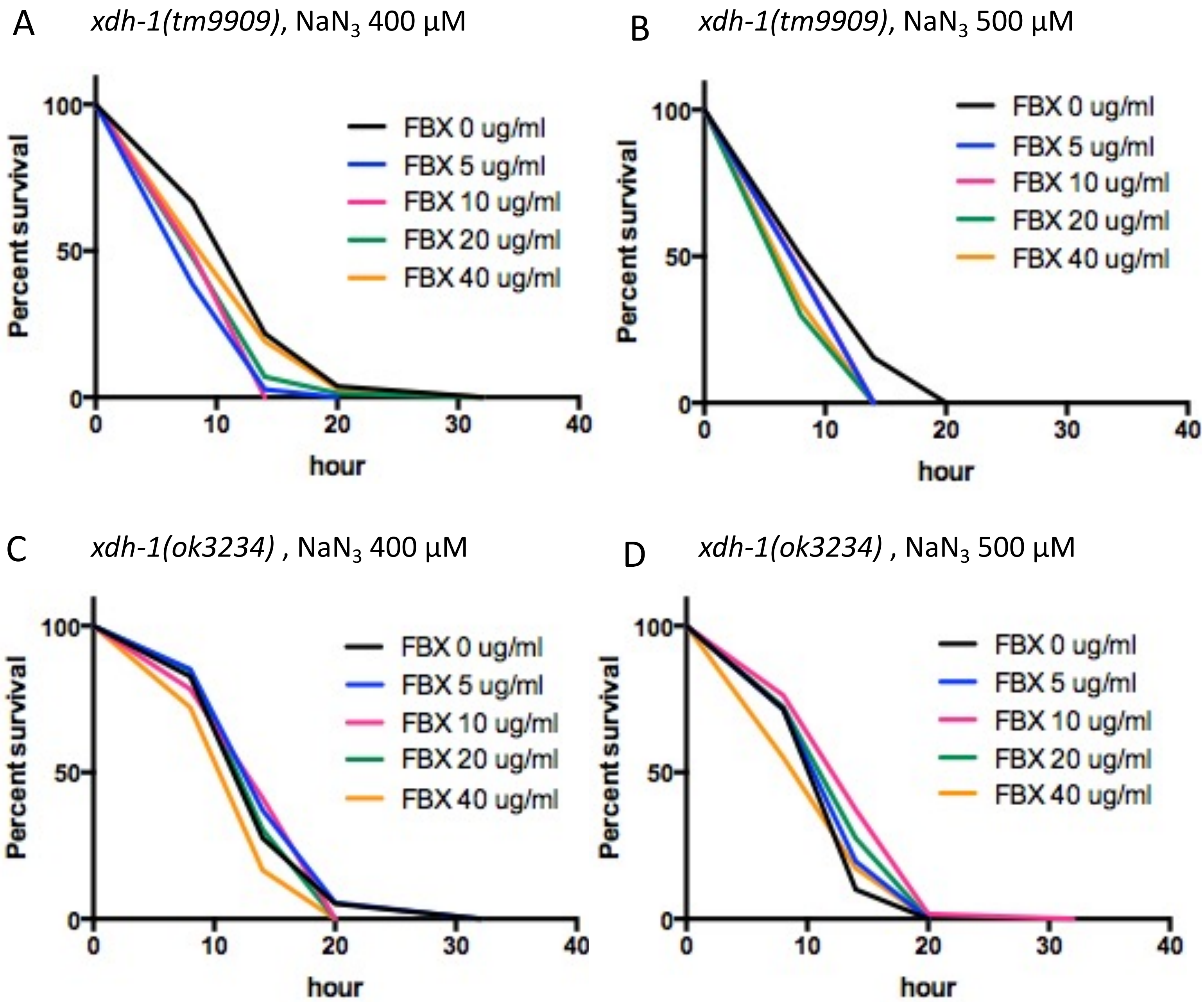

supplementary Fig. 6

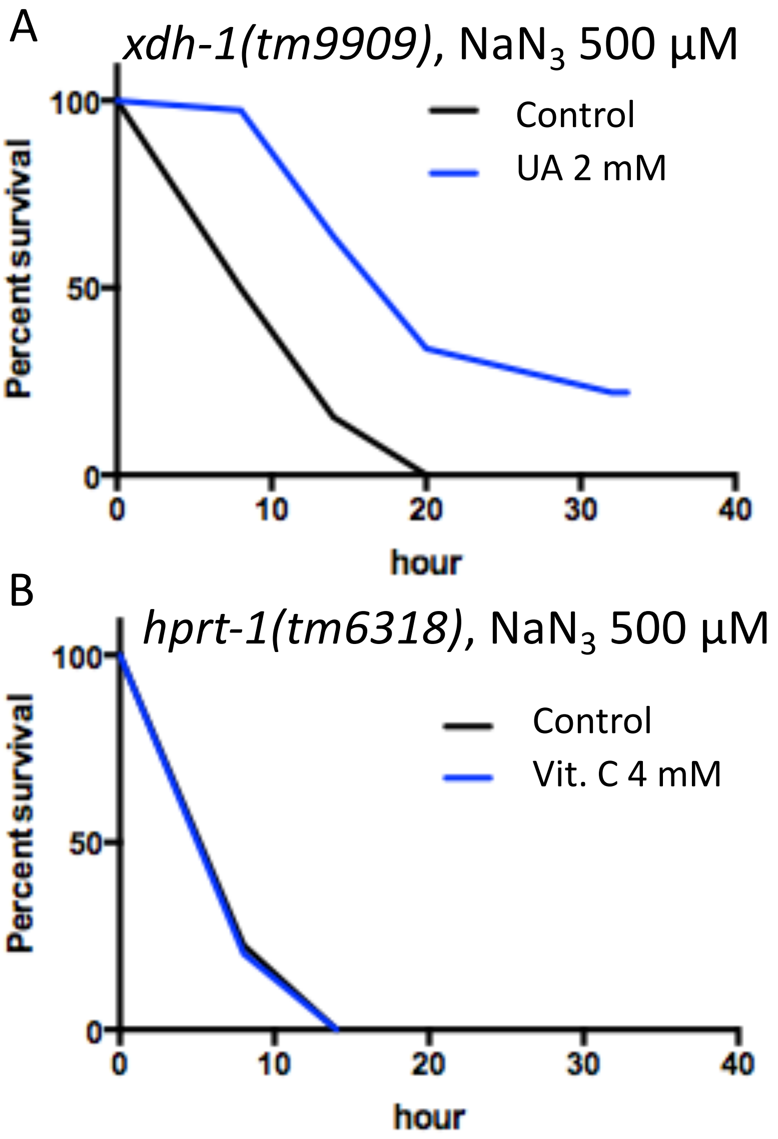

supplementary Fig. 7

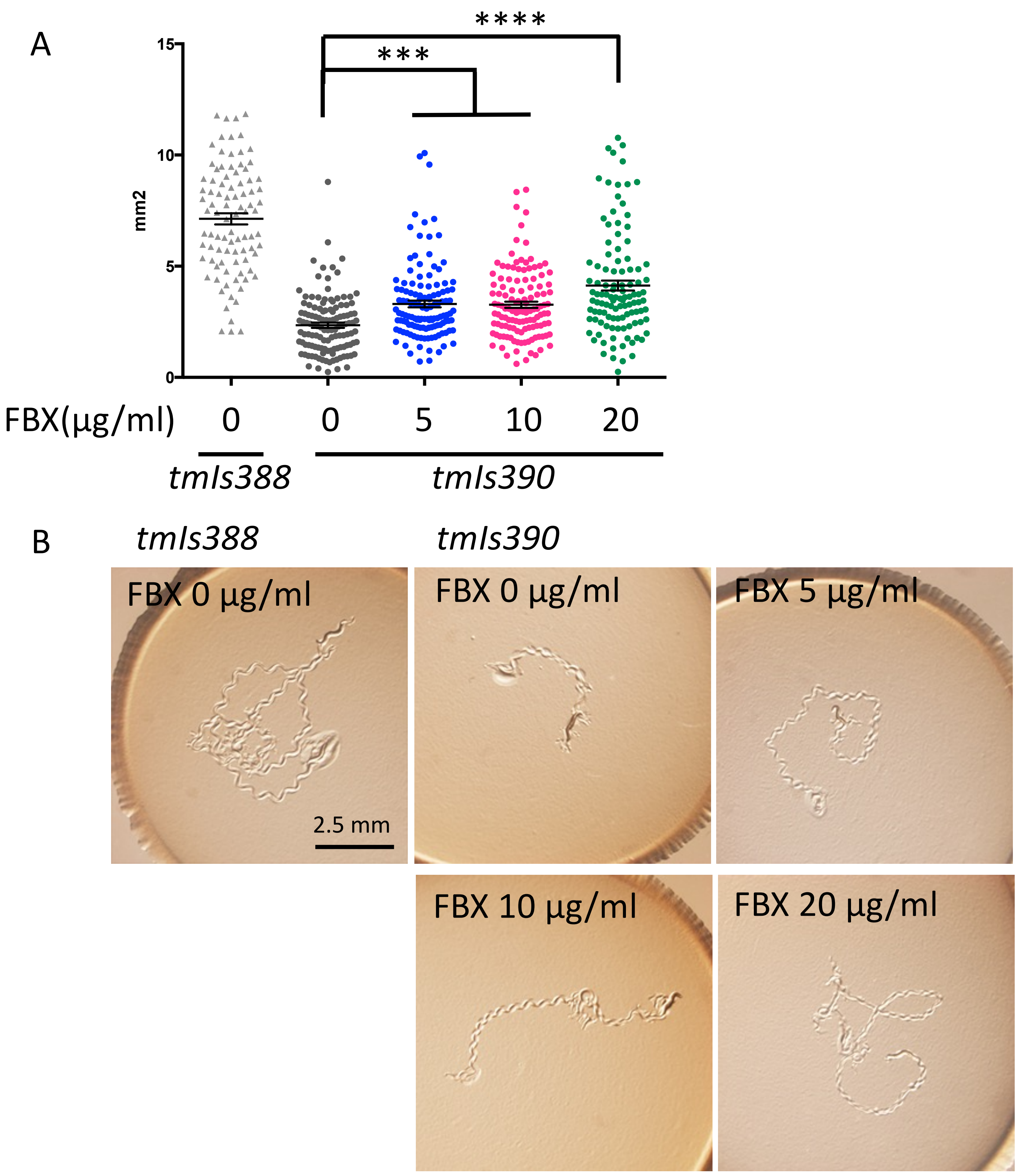

supplementary Fig. 8
